# A hormonal message for a longer life

**DOI:** 10.7554/eLife.113125

**Published:** 2026-10-22

**Authors:** Kyle Feola, Peter M Douglas

**Affiliations:** 1 Department of Molecular Biology, University of Texas Southwestern Medical Center Dallas United States

**Keywords:** longevity, hormones, dafachronic acid, mTOR, bile acids, *C. elegans*

## Abstract

The steroid hormone dafachronic acid may carry the effects of reduced mTOR activity between tissues to coordinate metabolism and longevity across the body.

**Related research article** Schilling K, Zaufel A, Morris KM, Löhrke A, Saini R, Knölker HJ, Moustafa T, Antebi A. 2026. mTOR regulates longevity through a bile-acid like hormonal mechanism and DHS-26/DHRS1. *eLife* **15**:RP111898. doi: 10.7554/eLife.111898.

Aging is a coordinated process across multiple tissues. Metabolic changes in one tissue can influence the health and function of distant organs, suggesting that signals travelling between cells coordinate organismal health and the pace of aging. Despite this, many pathways that regulate lifespan are studied primarily for their actions within individual cells and tissues. Identifying signals that convert local changes into organism-wide adaptations is therefore a key challenge in aging biology.

The protein kinase mTOR is a central regulator of metabolism, growth and aging. It is also a key mediator of inter-tissue crosstalk and links cellular growth to nutrient availability. When nutrients are abundant, mTOR complex 1 (mTORC1) promotes the production of new proteins and lipids while suppressing cellular recycling. When nutrients become scarce, mTORC1 activity is downregulated, shifting cells away from growth and toward maintenance and stress resistance.

It has been shown that reducing mTOR signaling extends lifespan in yeast, worms, flies and mice, placing it among the most reproducible interventions in aging biology (Vellai et al., 2003; Harrison et al., 2009). However, mTOR also supports growth, reproduction, immunity and tissue repair. The challenge is therefore not simply to inhibit mTOR, but to identify the downstream responses that produce its benefits while preserving the essential components of its activity. Now, in eLife, Adam Antebi and colleagues at research institutes in Germany and Austria – including Klara Schilling as first author – identify a compelling candidate for this long-range signal (Schilling et al., 2026).

Most explanations for mTOR-dependent longevity have focused on intracellular dynamics, including reduced protein synthesis, increased autophagy and altered mitochondrial metabolism. However, mTOR can also regulate aging cells indirectly. For example, in *Caenorhabditis elegans*, reducing a regulator of TORC1 in the nervous system, called RAGA-1, is sufficient to extend the lifespan of the whole animal. How this neuronal change is communicated to other tissues and affects the animal’s overall longevity has remained unclear.

Schilling et al. used a combination of lifespan experiments, gene and protein assays and microscopy to study the impact of RAGA-1 in wild-type and genetically modified *C. elegans*. The researchers found that longevity caused by reduced RAGA-1 activity (and thus reduced TORC1) requires a bile acid-like steroid hormone, dafachronic acid, and its dedicated nuclear receptor DAF-12. Because dafachronic acid can circulate between cells and activate transcription in distant tissues, it offers a plausible endocrine mechanism by which reduced mTOR activity in one location could reorganize physiology across the animal (Figure 1).

**Figure 1.**
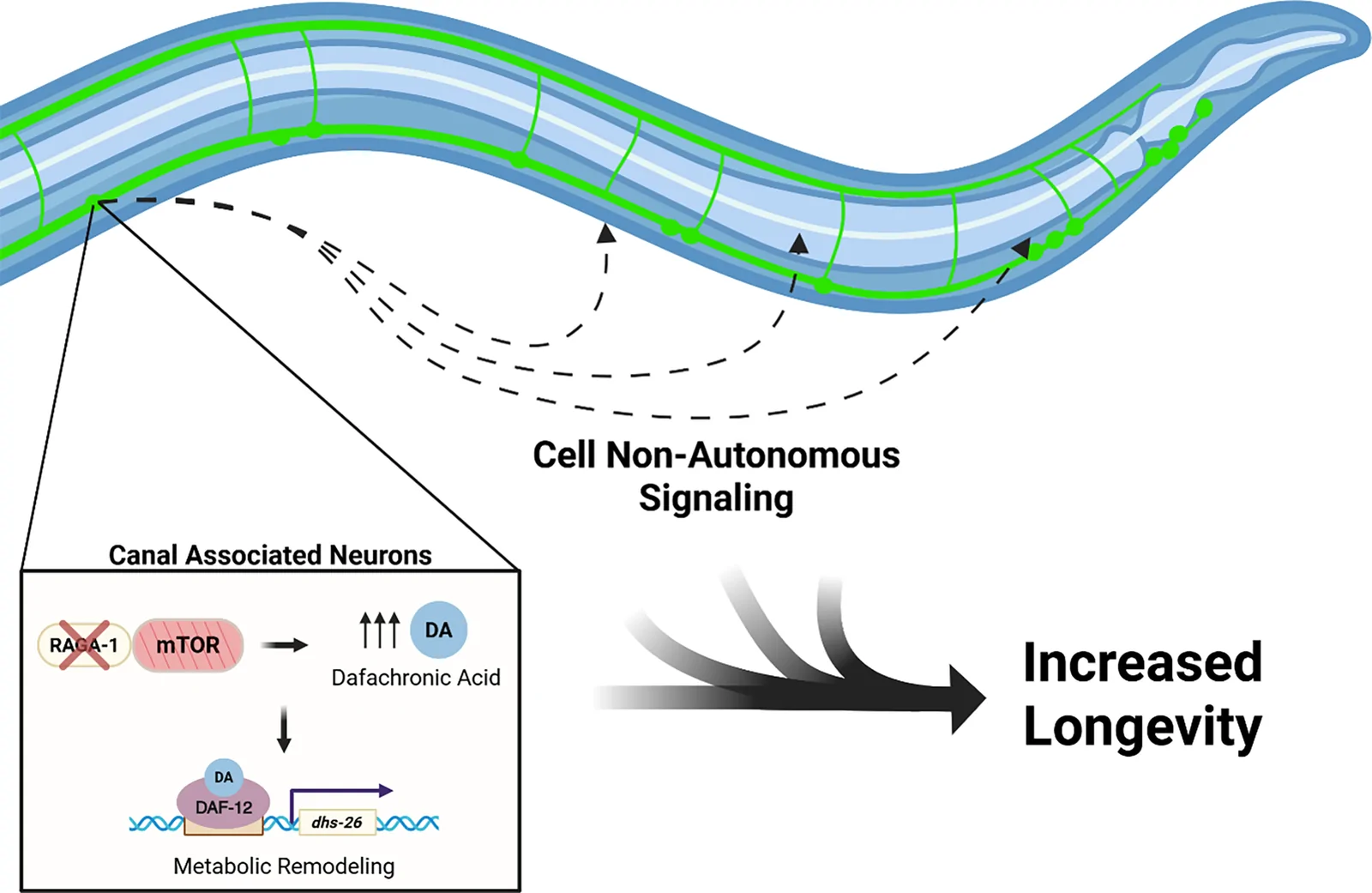
Endocrine regulation of longevity downstream of TORC1. Reduced RAGA-1–TORC1 signaling engages a DA–DAF-12–DHS-26 circuit that may communicate metabolic information from canal-associated neurons to unidentified target tissues, coordinating systemic metabolism and longevity.

This connection also brings the field full circle. Nearly 20 years ago, Antebi, together with researchers in the laboratories of the late David Mangelsdorf and Steven Kliewer, identified dafachronic acids as endogenous ligands for DAF-12, deorphanizing the first nuclear hormone receptor in *C. elegans* (Motola et al., 2006). That discovery showed how hormonal signaling couples environmental conditions to developmental fate. The study of Schilling et al. extends this principle by showing that the same endocrine machinery can translate nutrient sensing into longevity.

Schilling et al. revealed that disrupting either dafachronic acid production or DAF-12 prevents the lifespan extension normally caused by reduced RAGA-1/TORC1 signaling. Supplementing dafachronic acid restores longevity when hormone production is impaired, but not when its receptor is absent. Reduced TORC1 activity also increases endogenous dafachronic acid. Together, these findings place dafachronic acid/DAF-12 signaling downstream of mTOR and suggest that dafachronic acid carries its pro-longevity message across tissues.

The study also identified the enzyme DHS-26 as a critical component of this response. DHS-26 is required for the increased dafachronic acid and longevity associated with reduced TORC1 signaling, and in turn, increased dafachronic acid and DAF-12 activity promote DHS-26 expression. This reciprocal regulation could create a feedback circuit that amplifies an initially localized nutrient signal into a sustained, organism-wide metabolic program.

Intriguingly, DHS-26 is expressed prominently in the two canal-associated neurons (which support larval development), with weaker expression in a small number of head neurons and glial cells. Despite this restricted pattern, loss of DHS-26 abolished mTOR-dependent lifespan extension throughout the animal. These findings are consistent with a neuroendocrine model in which specialized cells regulate steroid hormone availability and thereby influence distant tissues. However, tissue-specific manipulation will be needed to establish precisely where DHS-26 acts in the animal to produce dafachronic acid and which tissues receive the DAF-12 signal.

The broader significance of the study lies in the cell non-autonomous activity of mTOR. In mammals, bile acids also function as endocrine hormones that signal through nuclear receptors, such as FXR, to coordinate metabolism among the intestine, liver and other organs. The mammalian DHS-26-related enzyme DHRS1 is regulated by both rapamycin and FXR-associated signaling, indicating that aspects of this pathway may be conserved in higher organisms.

The work of Schilling et al. suggests that reduced mTOR activity does not extend life solely by causing changes in individual cells, but also affects hormonal signaling throughout the organism, which tissues coordinate their metabolic priorities. Determining the source, destination and timing of this endocrine message and whether it can be selectively manipulated in mammals now represents an important direction for the field.

## References

[bib1] Harrison DE, Strong R, Sharp ZD, Nelson JF, Astle CM, Flurkey K, Nadon NL, Wilkinson JE, Frenkel K, Carter CS, Pahor M, Javors MA, Fernandez E, Miller RA (2009). Rapamycin fed late in life extends lifespan in genetically heterogeneous mice. Nature.

[bib2] Motola DL, Cummins CL, Rottiers V, Sharma KK, Li T, Li Y, Suino-Powell K, Xu HE, Auchus RJ, Antebi A, Mangelsdorf DJ (2006). Identification of ligands for DAF-12 that govern dauer formation and reproduction in *C. elegans*. Cell.

[bib3] Schilling K, Zaufel A, Morris KM, Löhrke A, Saini R, Knölker HJ, Moustafa T, Antebi A (2026). mTOR regulates longevity through a bile-acid like hormonal mechanism and DHS-26/DHRS1. eLife.

[bib4] Vellai T, Takacs-Vellai K, Zhang Y, Kovacs AL, Orosz L, Müller F (2003). Genetics: influence of TOR kinase on lifespan in *C. elegans*. Nature.

